# Effects of *APOE* gene ε4 allele on serum lipid profiles and risk of cardiovascular disease and tumorigenesis in southern Chinese population

**DOI:** 10.1186/s12957-022-02748-2

**Published:** 2022-09-03

**Authors:** Caiyan Gan, Yinmei Zhang, Fei Liang, Xuemin Guo, Zhixiong Zhong

**Affiliations:** 1 Center for Precision Medicine, Meizhou People’s Hospital, Meizhou, China; 2 Guangdong Provincial Key Laboratory of Precision Medicine and Clinical Translational Research of Hakka Population, Meizhou, China; 3 Guangdong Provincial Engineering and Technological Research Center for Molecular Diagnostics of Cardiovascular Diseases, Meizhou, China; 4 Data Center, Meizhou People’s Hospital, Meizhou, China

**Keywords:** Apolipoprotein E, Gene polymorphism, Tumor, Cardiovascular disease, Southern China

## Abstract

**Background:**

Human apolipoprotein E (APOE) polymorphisms are attributable to the presence of three common alleles, namely, ε2, ε3, and ε4, which generate six genotypes, viz, E2/E2, E2/E3, E3/E3, E3/E4, E4/E4, and E2/E4. *APOE* polymorphisms are associated with all types of tumors and cardiovascular diseases (CVD). However, the relationship between the type of *APOE* polymorphisms and tumorigenesis remains debatable. Therefore, we aimed to investigate the role of *APOE* polymorphisms on the tumor with or without CVD in southern China.

**Methods:**

A total of 1438 participants were categorized into 4 groups: 409 patients with tumor, 369 patients with CVD, 338 patients with both tumor and CVD, and 322 controls. *APOE* polymorphisms were determined by genotyping assay. The factors influencing tumor patients with or without CVD were also analyzed by logistic regression analysis.

**Results:**

The present study involved different types of solid tumors. Lung cancer was the most common cancer (20.2%, 151/747), followed by colorectal (17%, 127/747), esophageal (9.8%, 73/747), and liver (8.7%, 65/747) cancers. E3/E3 was the most frequent genotype, and ɛ3 was the greatest allele frequency in our study population. The frequencies of the E3/E3, E3/E4, E2/E3, E2/E4, E4/E4, and E2/E2 genotypes in tumor patients were 76.97% (575/747), 14.19% (106/747), 6.83% (51/747), 1.2% (9/747), 0.4% (3/747), and 0.4% (3/747), respectively. Tumor patients carrying ε3 with or without CVD showed higher levels of TG, TC, and LDL-C and lower levels of HDL-C compared to the controls carrying ε3. On the other hand, the tumor patients carrying ε4 with or without CVD showed higher levels of TG and LDL-C and lower levels of HDL-C (all *P* < 0.05). The frequency of *APOE* ε4 allele and the E3/E4 genotype was relatively greater in tumor or CVD patients (*P* < 0.001). In addition, ε4 allele acted as an independent risk factor for tumor patients group (*P* = 0.037, adjusted *OR* = 1.92, 95% *CI* 1.04–3.55) and tumor + CVD patients group (*P* = 0.012, adjusted *OR* = 2.53, 95% *CI* 1.22–5.23).

**Conclusions:**

Individuals carrying ε4 are at a higher risk of tumor with or without CVD, and *APOE* polymorphisms affect the serum lipid profiles.

## Introduction

Apolipoprotein E (APOE) is a multifunctional protein that plays a key role in the transport and metabolism of lipoprotein [1]. The human *APOE* is located on chromosome 19q13.32, contains 4 exons and 3 introns, and encodes a 34 KDa glycoprotein containing 299 amino acids [2]. *APOE* gene is polymorphic; composed of three common alleles, namely ε2, ε3, and ε4; and generates six different genotypes, including E2/E2, E2/E3, E2/E4, E3/E3, E3/E4, and E4/E4 [3]. E3/E3 genotype and ε3 allele were present most frequently in the human groups termed wild type of *APOE*, while E2/E2, E2/E3, E2/E4, E3/E4, E4/E4, ε2, and ε4 were considered the mutated forms [4]. Our past study results showed that the E3/E3 genotype accounted for approximately 65.43% of the Hakka population in southern China [5]. APOE is synthesized in the liver, brain, kidney, lungs, spleen, epidermis, and uterus [6]. Cardiovascular diseases (CVD) and cancer are the two main causes of death around the world [7]. The incidence rate of cancer and CVD is increasing worldwide which has an impact on the collective socioeconomic burden [8, 9]. Chronic inflammation is the pathogenesis of CVD and cancer [10, 11]. The role of inflammation in promoting carcinogenesis was established in the nineteenth century by Rudolf Virchow who then came up with a hypothesis [12]. Extensive factual evidence supports the relationship between inflammation and carcinogenesis through a complex interplay [13]. It has been reported that > 25% of all cancers are drawn by chronic inflammation [14]. Diabetes mellitus, obesity, dietary habits, physical activity, smoking habits, ethnicity, age, gender, and genetic differences are some of the risk factors of both CVD and cancer [15].

Dyslipidemia is a significant risk factor for cancer. Muntoni et al. found that the serum high-density lipoprotein cholesterol (HDL-C) and apolipoprotein A1 (ApoA1) levels of tumor patients were lower, and that their triglyceride (TG) levels were higher (all *P* < 0.05) than those of the control subjects [16]. A past study reported that the serum levels of total cholesterol (TC) and low-density lipoprotein cholesterol (LDL-C) were positively correlated with the occurrence of colorectal cancer [17]. It is also well known that dyslipidemia has major implications for the risk of CVD [18, 19]. *APOE* polymorphism has been associated with CVD [20]. Different APOE isoforms have been reported to be associated with a significant variation in the patients’ lipid profiles [21]. Recently, it was established that altered serum lipoprotein profile has diagnostic and prognostic significance for cancer when atherosclerosis and inflammatory diseases can be ruled out [16]. Thus, it is possible that genetic factors that affect the regulation and metabolism of lipids also influence the degree of susceptibility to tumors and CVD. In addition, previous literatures have demonstrated that CVD and cancer share risk factors and biological mechanisms [15, 22]. A recent study composed of 8592 subjects evaluated whether tumor biomarkers can predict new-onset CVD and mortality in the Prevention of Renal and Vascular End-stage Disease (PREVEND) cohort; the findings supported the notion that CVD and cancer are associated with similar pathological milieus [23]. On the other hand, several studies have hinted toward an association between CVD and cancer incidence and mortality [24, 25].

Several past studies have reported that the ε4 allele is an independent genetic risk factor for coronary artery disease (CAD) [26], age-related Alzheimer’s disease (AD) [27], type 2 diabetes mellitus (T2DM) [28], and CVD [29]. Furthermore, the ε2 allele has also been associated with increased CVD or T2DM risk [30, 31]. In addition, *APOE* polymorphisms also mediate the incidence of breast cancer, colorectal cancer, prostate cancer, gastric cancer, endometrial carcinoma, pituitary adenoma, and head and neck cancer [32–39]. However, the published data on associations between *APOE* polymorphism and cancer risk were highly inconsistent. Chang et al. revealed that the *APOE* ε4 allele was significantly associated with increased risk of tumorigenesis in breast cancer in the Taiwanese population [32]. In contrast, Moysich et al. reported no significant association between ε4 allele variant and breast cancer [40]. Another study reported that the presence of ε2 allele and ε4 allele was positively associated with breast cancer in Brazil [41]. On the other hand, a study in the Chinese population demonstrated that the ε2 allele was a risk factor for gastric cancer [42], while De Feo et al. noted that the ε2 allele significantly decreased the risk for gastric cancer risk [34]. Some other studies have reported that the ε4 allele decreased the risk for colorectal cancer [43, 44], while some have reported that the presence of ε4 allele was not associated with colorectal cancer risk [45]. A meta-analysis of 8 studies, which included 4310 colorectal neoplasia (CRN) cases and 4933 healthy controls from the Asians, Brazilian, Chinese, and Caucasians population, indicated that the *APOE* ɛ4 allele was associated with a decreased risk of proximal CRN, except for distal CRN [46]. It has also been reported that ε2 carriers tend to be low levels of plasma cholesterol and LDL-C, whereas ε4 carriers tend to have higher levels of LDL-C [47]. Evidences from several studies demonstrate that individuals with low serum LDL-C and TC levels significantly increased cancer risk [34, 48]. A past study reported that the presence of the ε4 allele is relatively more associated with an increased risk of CVD and tumor occurrence than that of the non-ε4 allele carriers [49]. Furthermore, to the best of our knowledge, there are no literature reporting any relationship between *APOE* polymorphisms and the risk of CVD and cancer in the southern China population.

Through this study, we aimed to investigate whether different *APOE* genotypes affect CVD and cancer in a southern China population by exploring the possible association of *APOE* polymorphisms between CVD and cancer patients.

## Methods

### Study population

A total of 1438 individuals were recruited from the inpatients of Meizhou People’s Hospital, Guangdong, China, between May 2016 and September 2020. The recruits included 409 tumor patients, 369 CVD patients, 338 CVD + tumor patients, and 322 control subjects (non-CVD and non-tumor). CVD or tumor patients were diagnosed by professional clinicians based on the related clinical symptoms, history, pathology, and laboratory and imaging findings. All subject demographics were recorded, including age, blood pressure, gender, alcohol intake, smoking habit, *APOE* genotyping, lipid profile, hypertension, diabetes, dyslipidemia, and fatty liver. Hypertension was defined as currently undertaking treatment with a blood pressure medication or SBP/DBP level ≥ 140/90 mmHg. Dyslipidemia was defined as lipid profile meeting any one of the following conditions: serum level of triglycerides (TG) > 1.7 mmol/L, TC > 5.5 mmol/L, LDL-C > 3.1 mmol/L, and HDL-C < 0.88 mmol/L. Diabetes mellitus was defined as a fasting blood glucose level ≥ 6.67 mmol/L or non-fasting glucose levels ≥ 11.11 mmol/L or patients who were currently undergoing treatment with insulin or antidiabetic medications. The fatty liver diagnosis was based on the guideline of the American Association for the Study of Liver Diseases (AASLD) [50]. The exclusion criteria included the presence of uterine fibroids, meningioma, renal hamartoma, among other signs of benign tumor.

The present study protocol was approved by the Ethics Committee at Meizhou People’s Hospital (No.: 2018-C-12) and conducted in accordance with the principles of the 1975 Declaration of Helsinki. All participants provided their signed informed consent forms before participating in the study.

### DNA extraction and genotyping

From all participants, 2 mL of the fasting venous blood samples was collected and stored into an ethylenediaminetetraacetic acid (EDTA) tube containing anticoagulants. Genomic DNA was extracted from blood using the Blood DNA Isolation Kit (Tiangen Biotech, Guangdong, China). DNA concentration and quality were evaluated using the Nano-Drop 2000 Spectrophotometer (ThermoFisher Scientific). TaqMan probe fluorescent polymerase chain reaction (PCR) method was employed for *APOE* genotyping using different probes and primer combinations. The PCR forward primer and reverse primer sequences were as follows: 5′-GCTTGGCACGGCTGTCCAAGGA-3′ and 5′-ATTCGCCCCGGCCTGGTACAC-3′, respectively. PCR was performed by amplifying the target fragments under the following conditions: 50 °C for 2 min, 95 °C for 15 min, 94 °C for 30 s (amplification of 45 cycles), and 65 °C for 45 s. Agarose gel electrophoresis was performed to confirm the amplified product of *APOE* after PCR. The amplification product was subsequently added to a hybridization reaction gene chip assay (Zhuhai Sinochips Bioscience Co., Ltd., Guangdong, China), and the enzymatic chromogenic reaction revealed the color of the specific hybridization signal. To validate the results, blank control, negative control, and positive control were included in all *APOE* gene SNPs chip assays. In addition, to confirm the quality and accuracy of genotyping data from the gene chip assay, Sanger sequencing was also randomly carried out in the 10% duplicate samples in this study.

### Biochemical measurements

Approximately, 3 mL of fasting blood sample from each subject was collected in the morning after an 8 to 12 h of overnight fast and transfused into vacuum tubes without an anticoagulant. The serum was rapidly separated and evaluated using the Olympus AU5400 system (Olympus Corporation, Tokyo, Japan) for the concentrations of TG, TC, LDL-C, and HDL-C. The fasting lipid profiles were measured in accordance with the manufacturers’ instructions.

### Statistical analysis

SPSS statistical software version 21.0 (IBM Inc., State of New York, USA) was used for data analyses. Kolmogorov-Smirnov test was performed to evaluate data normality. Continuous data were reported as the mean ± standard deviation (SD) or median (interquartile) based on the data normality of distribution. Categorical variables were represented by frequency. Groups of continuous data analysis were performed with Mann-Whitney *U*-test. The allele (ε2, ε3, ε4) and genotype (E2/E2, E2/E3, E2/E4, E3/E3, E3/ E4, E4/E4) are respectively of the control group (without CVD or tumor) as a reference [51–53]. Chi-square test was performed for comparing allele frequencies composition ratios. The Fisher’s exact and chi-square tests were employed to compare the genotype composition ratios. When the sample number was ≥ 40 and the theoretical frequency was ≥ 5, the chi-square test was used; otherwise, the Fisher’s exact test was used to compare genotype composition ratios. Logistic regression analysis was performed to assess the association among different types of diseases and the risk factors with the adjusted odds ratio (OR).

## Results

### Population clinical characteristics

Table 1 lists the baseline clinical characteristics of all participants. A total of 1438 participants were enrolled in this study, who were assigned into the following 4 groups: control (*n* = 322), tumor (*n* = 409), CVD (*n* = 369), and tumor + CVD (*n* = 338), respectively. The mean age was more than 60 years in all four groups, with a statistically significant difference between the control and the CVD or the CVD + tumor. A significant gender difference was also observed among the four groups, with a higher prevalence among men. The levels of SBP, DBP, TC, TG, and LDL-C were significantly higher in the patients (*P* < 0.05), while the level of HDL-C was lower in the tumor and CVD + tumor groups (*P* < 0.001) compared to that in the control group. The number of cigarettes smoked by CVD patients (*P* < 0.01) and CVD + tumor patients (*P* < 0.05) was more than that of the control subjects. The incidence of dyslipidemia, hypertension, diabetes, and fatty liver was higher in the CVD + tumor groups than in the tumor or CVD group. The prevalence of dyslipidemia, hypertension, and diabetes was 26, 56.8, and 34.3%, respectively, in the CVD + tumor group: 12, 28.4, and 23.5%, respectively, in the tumor group and 5.4, 53.1, and 19.5%, respectively, in the CVD group.

**Table 1:** Characteristics of the study population

	Control (*N* = 322)	Tumor (*N* = 409)	CVD (*N* = 369 )	CVD + tumor (*N* =338)
Age (years)	61.53 ± 12.67	63.33 ± 12.16	64.96 ± 11.29**	69.89 ± 10***
SBP (mmHg)	124 (16.3)	133 (27)***	135 (16)***	139.5 (30)***
DBP (mmHg)	76 (11)	80 (14)***	83 (10)***	80.5 (18)***
Male/female (%)	179/143 (55.6%/44.4%)	260/149 (63.6%/36.4%)*	236/133 (64%/36%)*	243/95 (71.9%/28.1%)***
Smoking (%)	68 (21.1%)	87 (21.3%)	115 (31.2%)**	96 (28.4%)*
Drinking (%)	23 (7.1%)	45 (11%)	23 (6.2%)	28 (8.3%)
Hypertension (%)	/	116 (28.4%)	196 (53.1%)	192 (56.8%)
Diabetes (%)	/	96 (23.5%)	72 (19.5%)	116 (34.3%)
Dyslipidemia (%)	/	49 (12%)	20 (5.4%)	88 (26%)
Fatty liver (%)	/	29 (7.1%)	36 (9.8%)	41 (12.1%)
TC (mmol/L)	4.53 (0.56)	4.65 (1.64)**	4.94 (0.59)***	4.69 (1.72)**
TG (mmol/L)	0.93 (0.5)	1.24 (0.77)***	1.14 (0.51)***	1.27 (1.04)***
LDL-C (mmol/L)	2.5 (0.42)	2.62 (1.13)***	2.81 (0.49)***	2.68 (1.21)***
HDL-C (mmol/L)	1.38 (0.34)	1.19 (0.47)***	1.38 (0.27)	1.18 (0.48)***

Data are presented as median (interquartile range) or mean ± standard deviation, numbers (percentage). *P < 0.05, **P < 0.01, ***P < 0.001: comparison with control. *SBP* systolic blood pressure, *DBP* Diastolic blood pressure, *TC* Total cholesterol, *TG* Triglyceride, *LDL-C* Low-density lipoprotein cholesterol, *HDL-C* high-density lipoprotein cholesterol, *CVD* Cardiovascular disease

### Genotype and allele frequencies of APOE

The genotype distribution of all groups was consistent with the Hardy-Weinberg equilibrium (*P* > 0.05). E3/E3 was the most frequent genotype, while ɛ3 was the greatest allele frequency (Table 2) in our study population. When compared with the control group by using chi-square test to estimate the risk of each of the ApoE alleles and the genotypes in the tumor and CVD groups, the frequency of ε3 and E3/E3 was significantly decreased in the tumor patients with or without CVD group (*P* < 0.01), while those of the ε4 and E3/E4 were significantly increased (*P* < 0.001), suggesting a a risk factor ε4 in tumor and CVD genesis. In addition, no statistically significant differences were noted in the E2/E2, E2/E3, E2/E4, and E4/E4 genotypes and the ε2 allele between the control group and the tumor groups with or without CVD (Table 2). The corresponding details are summarized in Table 2.

**Table 2:** Alleles and genotypes distribution and the risk of APOE polymorphism in different groups

	Control (*N* = 322)	Tumor (*N* = 409)	Unadjusted OR (95% *CI*)	CVD (*N* = 369)	Unadjusted OR (95% *CI*)	CVD + tumor (*N* = 338)	Unadjusted OR (95% *CI*)
Allele							
ε2	25 (3.9%)	38 (4.6%)	1.21 (0.72–2.02)	45 (6.1%)	1.61 (0.97–2.65)	28 (4.1%)	1.07 (0.62–1.86)
ε3	595 (92.4%)	716 (87.5%)	0.58 (0.4–0.83)**	605 (82%)	0.38 (0.27–0.53)***	591 (87.4%)	0.57 (0.4–0.83)**
ε4	24 (3.7%)	64 (7.8%)	2.19 (1.36–3.55)***	88 (11.9%)	3.5 (2.2–5.57)***	57 (8.4%)	2.38 (1.46–3.88)***
Total	644	818		738		676	
Genotype							
E2/E2	1 (0.3%)	1 (0.2%)	0.79 (0.05–12.63)	2 (0.5%)	1.75 (0.16–19.38)	2 (0.6%)	1.91 (0.17–21.18)
E2/E3	20 (6.2%)	29 (7.1%)	1.15 (0.64–2.08)	35 (9.5%)	1.58 (0.89–2.8)	22 (6.5%)	1.05 (0.56–1.97)
E2/E4	3 (0.9%)	7 (1.7%)	1.85 (0.48–7.22)	6 (1.6%)	1.76 (0.44–7.09)	2 (0.6%)	0.63 (0.11–3.81)
E3/E3	278 (86.3%)	317 (77.5%)	0.55 (0.37–0.81)**	246 (66.7%)	0.32 (0.22–0.47)***	258 (76.3%)	0.51 (0.34–0.77)***
E3/E4	19 (5.9%)	53 (13%)	2.37 (1.38–4.1)***	78 (21.1%)	4.28 (2.53–7.24)***	53 (15.7%)	2.97 (1.71–5.13)***
E4/E4	1 (0.3%)	2 (0.5%)	1.59 (0.14–17.47)	2 (0.5%)	1.75 (0.16–19.38)	1 (0.3%)	0.95 (0.06–15.29)
Total	322	409		369		338	
HDW	*P* = 0.15	*P* = 0.18		*P* = 0.56		*P* = 0.31	

Data are presented as numbers (percentage), *HDW* Hardy-Weinberg equilibrium, *P < 0.05: comparison with control, *CI* confidence interval

### Association of APOE polymorphism and tumor

The type, gender, and frequency distribution of malignant tumors and *APOE* genotype are presented in Tables 3 and 4. A total of 747 (244 women, 503 men) patients of the tumor group and CVD + tumor group were enrolled. Lung cancer was the most common cancer among the patients (20.2%, 151/747), followed by colorectal (17%, 127/747), esophagus (9.8%, 73/747), and liver (8.7%, 65/747) cancers. Notably, the tumor types were different between the genders. In men, the top 3 cancers based on their frequencies were lung (23.7%, 119/503), colorectal (17.1%, 86/503), and liver (10.9%, 55/503) cancers, while the corresponding top 3 cancers in women were colorectal (16.8%, 41/244), lung (13.1%, 32/503), and breast (11.5%, 21/244) cancers. In the 747 tumor patients, the frequencies of E3/E3, E3/E4, E2/E3, E2/E4, E4/E4, and E2/E2 genotypes were 76.97% (575/747), 14.19% (106/747), 6.83% (51/747), 1.2% (9/747), 0.4% (3/747), and 0.4% (3/747), respectively.

**Table 3:** Tumor types in 747 women or men patients

Type	Number (%)		
	Men (*N* = 503)	Women (*N* = 244)	Total (*N* = 747)
Lung cancer	119 (23.7%)	32 (13.1%)	151 (20.2%)
Colorectal cancer	86 (17.1%)	41 (16.8%)	127 (17%)
Esophagus cancer	52 (10.3%)	21 (8.6%)	73 (9.8%)
Liver cancer	55 (10.9%)	10 (4.1%)	65 (8.7%)
Nasopharynx cancer	31 (6.2%)	8 (3.3%)	39 (5.2%)
Pituitary tumor	15 (3%)	20 (8.2%)	35 (4.7%)
Stomach cancer	21 (4.2%)	11 (4.5%)	32 (4.3%)
Breast cancer	/	28 (11.5%)	28 (3.7%)
Neurologic tumor	16 (3.2%)	11 (4.5%)	27 (3.6%)
Prostatic cancer	24 (4.8%)	/	24 (3.2%)
Hematological malignancies	14 (2.8%)	9 (3.7%)	23 (3.1%)
Cervical cancer	/	20 (8.2%)	20 (2.7%)
Bladder cancer	16 (3.2%)	1 (0.4%)	17 (2.3%)
Thyroid cancer	3 (0.6%)	8 (3.3%)	11 (1.5%)
Renal cancer	6 (1.2%)	1 (0.4%)	7 (0.9%)
Laryngocarcinoma	6 (1.2%)	/	6 (0.8%)
Ovarian cancer	/	6 (2.5%)	6 (0.8%)
Gastrointestinal stromal tumor	3 (0.6%)	2 (0.8%)	5 (0.7%)
Pancreatic cancer	3 (0.6%)	2 (0.8%)	5 (0.7%)
Thymic carcinoma	2 (0.4%)	2 (0.8%)	4 (0.5%)
Adrenal tumor	3 (0.6%)	/	3 (0.4%)
Endometrial cancer	/	3 (1.2%)	3 (0.4%)
More than two cancers	22 (10.9%)	4 (1.6%)	26 (3.5%)
Others	6 (1.2%)	4 (1.6%)	10 (1.3%)

**Table 4:** APOE genotype and tumor types

Type	Numbers (*N* = 747)					
	E2/E2 (*N* = 3)	E2/E3 (*N* = 51)	E2/E4 (*N* = 9)	E3/E3 (*N* = 575)	E3/E4 (*N* = 106)	E4/E4 (*N* = 3)
Lung cancer	1	14	2	102	31	1
Colorectal cancer	1	2	2	100	21	1
Esophagus cancer	/	5	1	58	9	/
Liver cancer	/	7	1	49	8	/
Nasopharynx cancer	/	3	/	30	6	/
Pituitary tumor	/	2	/	30	3	/
Stomach cancer	/	3	1	25	3	/
Breast cancer	/	2	/	20	6	/
Neurologic tumor	/	3	1	21	2	/
Prostatic cancer	/	/	/	10	4	/
Hematological malignancies	1	/	/	21	1	/
Cervical cancer	/	2	/	15	3	/
Bladder cancer	/	1	/	16	/	/
Thyroid cancer	/	/	1	9	1	/
Renal cancer	/	/	/	5	2	/
Laryngocarcinoma	/	/	/	5	1	/
Ovarian cancer	/	2	/	4	/	/
Gastrointestinal stromal tumor	/	/	/	5	/	/
Pancreatic cancer	/	1	/	4	/	/
Thymic carcinoma	/	/	/	3	1	/
Adrenal tumor	/	1	/	2	/	/
Endometrial cancer	/	1	/	1	1	/
More than two cancers	/	1	/	21	3	1
Others	/	1	/	9	/	/

### Relationships between APOE allele and lipid profiles

**Fig. 1:**
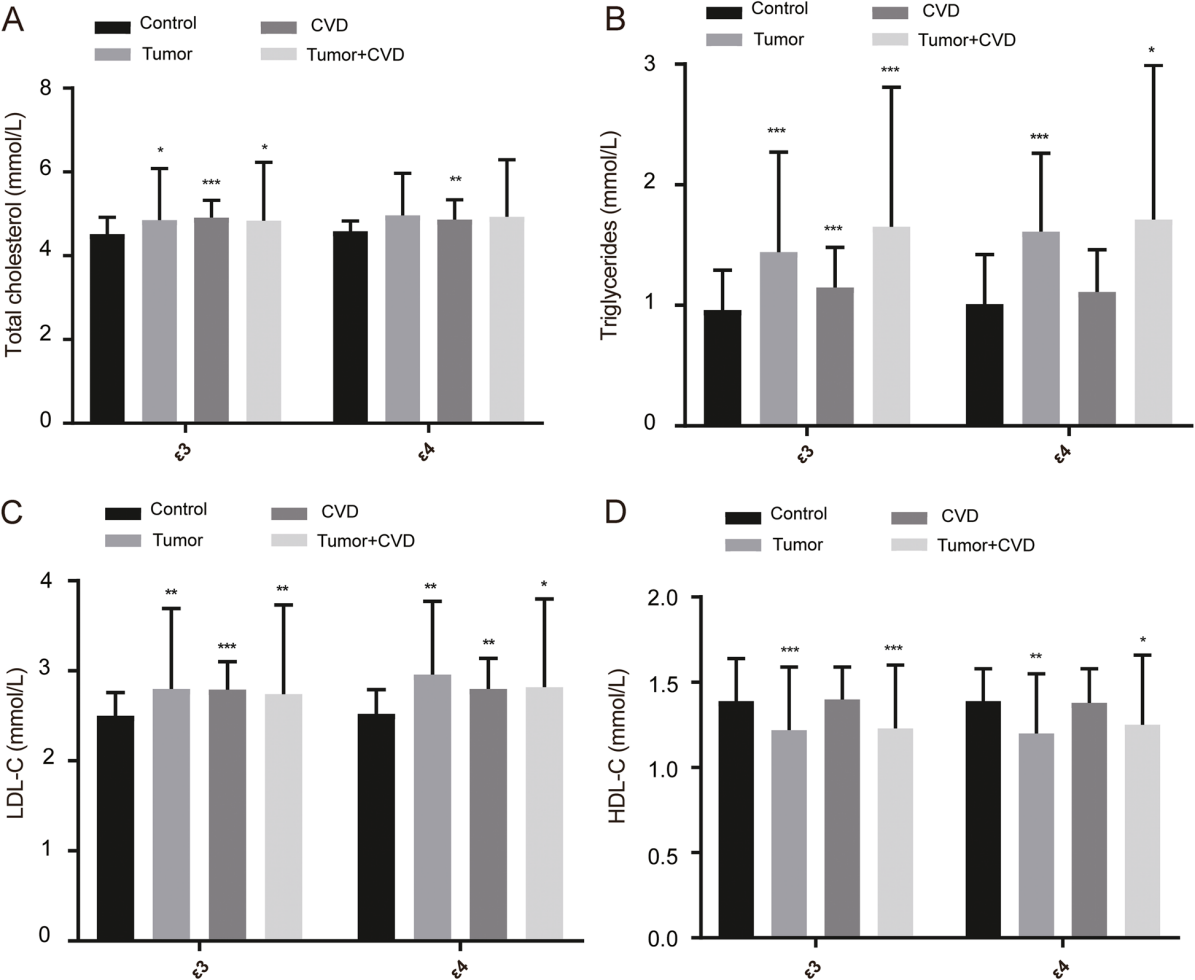
The lipid profile between subjects carrying ε3 and ε4 allele. **A** The levels of total cholesterol subjects carrying ε3 and ε4 allele. **B** The levels of triglycerides subjects carrying ε3 and ε4 allele. **C** The levels of LDL-C subjects carrying ε3 and ε4 allele. **D** The levels of HDL-C subjects carrying ε3 and ε4 allele. *ε*3 = E3/E3, *ε*4 = E3/E4 + E4/E4. *P < 0.05, comparison with ε3 and ε4 allele in control group respectively

We analyzed the relationships between the *APOE* alleles (i.e., ε3 and ε4) and the blood lipid levels (Fig. 1). In this study, ε3 and ε4 of the control group served as a reference. The ε3-carrier tumor patients with or without CVD showed higher levels of TG, TC, and LDL-C (*P* < 0.05) and lower levels of HDL-C (*P* < 0.001). Similarly, ε4-carrier tumor patients showed higher levels of TG (*P* < 0.05) and LDL-C (*P* < 0.05) but lower levels of HDL-C (*P* < 0.05). In the ε4-carrier tumor patients, the TC level was higher than those of the controls, albeit there were no significant differences (all *P* > 0.05). Meanwhile, in the meantime, the ε4-carrier tumor patients showed lower levels of HDL-C than ε4 of the control group (all *P* < 0.05).

### Logistic regression analysis of factors affecting tumor patients

A multiple logistic regression analysis was performed to evaluate the independent predictors for tumor patients, by adjusting the conventional factors including ε2, ε3, ε4, gender, age, smoking, TG, TC, LDL-C, HDL-C, SBP, and DBP. The tumors in this study were classified as tumor of the digestive system, respiratory system, reproductive system, endocrine system, nervous system, urinary system, and circulatory system according to different systems of the human body. After adjustment for the variables, ε4 allele served as an independent significant risk factors for digestive system tumor patients’ group (*P* = 0.049, adjusted *OR* = 2.19, 95% *CI* 1.0–4.76) and digestive system tumor + CVD patients’ group (*P* = 0.039, adjusted *OR* = 2.77, 95% *CI* 1.05–7.3), as well as for respiratory system tumor patients’ group (*P* = 0.01, adjusted *OR* = 2.93, 95% *CI* 1.29–6.63) and respiratory system tumor + CVD patients’ group (*P* = 0.007, adjusted *OR* = 4.15, 95% *CI* 1.48–11.7) (Table 5).

**Table 5:** Logistic regression analysis of APOE gene polymorphisms for different tumors

		Tumor versus control			Tumor + CVD versus control		
	Allele	Adjusted OR	95% *CI*	*p*-value	Adjusted OR	95% *CI*	*p*-value
Digestive system	ε2	1.6	0.71–3.6	0.26	1.22	0.39–3.78	0.73
	ε3	0.14	0.02–1.27	0.08	0.11	0.004–2.81	0.18
	ε4	2.19	1.0–4.76	0.049	2.77	1.05–7.3	0.039
Respiratory system	ε2	1.89	0.71–5.01	0.20	1.35	0.41–4.48	0.63
	ε3	0.17	0.02–1.89	0.15	1.08	0.01–82.23	0.97
	ε4	2.93	1.29–6.63	0.01	4.15	1.48–11.7	0.007
Reproductive system	ε2	0.41	0.06–3.0	0.38	0.35	0.03–4.44	0.42
	ε3	-	-	-	-	-	-
	ε4	0.80	0.12–5.34	0.82	4.51	0.87–23.45	0.07
Endocrine system	ε2	3.13	0.50–19.67	0.22	0.25	0.01–5.37	0.38
	ε3	0.04	0.00–3.98	0.17	-	-	-
	ε4	0.74	0.07–7.73	0.80	2.97	0.37–23.84	0.31
Nervous system	ε2	3.19	0.29–34.52	0.34	-	-	-
	ε3	-	-	-	-	-	-
	ε4	0.76	0.01–59.76	0.9	-	-	-
Urinary system	ε2	-	-	-	-	-	-
	ε3	-	-	-	-	-	-
	ε4	2.53	0.19–33.37	0.48	3.9	0.45–33.61	0.22
Circulatory system	ε2	0.92	0.08–10.78	0.95	-	-	-
	ε3	0.01	0.000–0.63	0.03	-	-	-
	ε4	-	-	-	-	-	-

Adjusted OR Adjusting the traditional factors including gender, age, smoking, TG, TC, LDL-C, HDL-C, SBP, and DBP

**Fig. 2:**
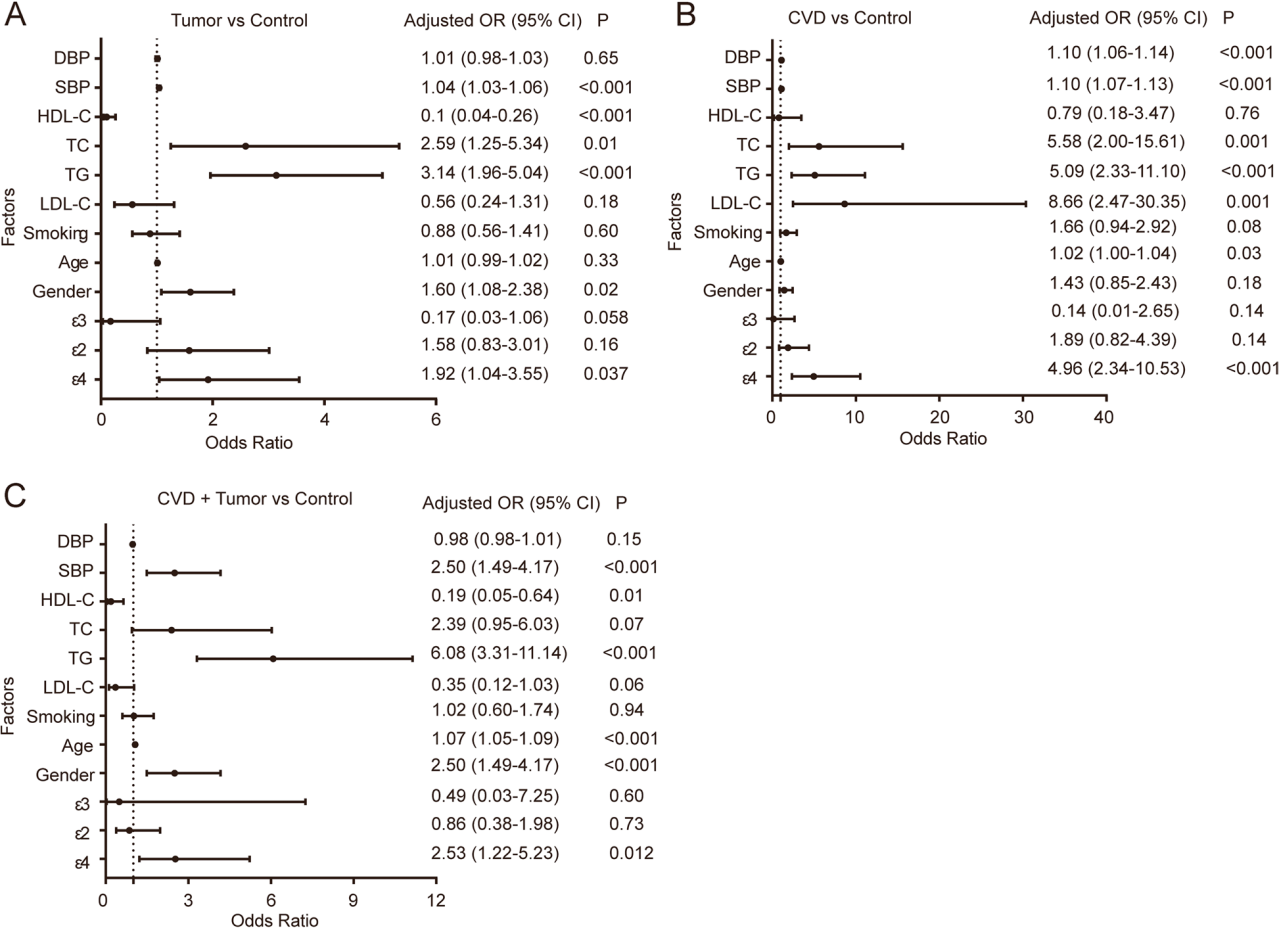
Logistic regression analysis of factors that influence tumor, CVD, and CVD + tumor patients. **A** Logistic regression analysis of factors for tumor patients. **B** Logistic regression analysis of factors for CVD. **C** Logistic regression analysis of factors for CVD + tumor patients

After adjustment for the quantitative variables, ε4 allele served as an independent significant risk factors for both the tumor patients’ group (*P* = 0.037, adjusted *OR* = 1.92, 95% *CI* 1.04–3.55), the CVD patients’ group (*P* < 0.001, adjusted *OR* = 4.96, 95% *CI* 2.34–10.53), and tumor + CVD patients’ group (*P* = 0.012, adjusted OR = 2.53, 95% CI 1.22-5.23) (Fig 2). The TG was an independent and significant risk factor for the tumor patients’ group, CVD patients’ group and tumor + CVD patients’ group (all *P* < 0.001). The TC increased the risk for tumor patients (*P* = 0.01, adjusted *OR* = 2.59, 95% *CI* 1.25–5.34) and the CVD patients’ group (*P* = 0.001, adjusted *OR* = 5.58, 95% *CI* 2.00-15.61). In addition, the risk factors for the tumor patients’ group also included SBP (*P* < 0.001), while those for the tumor + CVD patients’ group included SBP, age, and gender (all *P* < 0.05). However, HDL-C was found to further decrease the risk for tumor patients with or without CVD (all *P* < 0.05).

## Discussion

The common shared biological mechanisms and the risk factors may explain the association between cancer and CVD. Smoking, alcoholism, T2DM, obesity, physical inactivity, dyslipidemia, and genetic differences are shared risk factors common to both cancer and CVD. Pathways related to inflammation, metabolic remodeling, hypoxia, angiogenesis, clonal hematopoiesis, and the extracellular vesicles and circulating noncoding RNAs (ncRNAs) are shared pathophysiology for cancer and CVD [54, 55]. Epidemiological data has reported an increased cancer risk in patients with CVD [56]. In addition, the Women’s Health Study (WHS) demonstrated that 10% of patients who had new-onset atrial fibrillation (AF) developed subsequent cancer [57]. Increasing evidence suggests numerous commonalities in pathophysiologic mechanisms, and relationship exists between cancer and CVD. The tumor biomarker levels of cancer antigen 15-3 (CA 15-3), cancer antigen 19-9 (CA 19-9), carcinoembryonic antigen (CEA), cytokeratin fragment 21-1 (CYFPRA 21-1), and alpha-fetoprotein (AFP) were significantly higher in individuals with CVD when compared to individuals without CVD [23]. These tumor biomarkers revealed independent prognostic value for CVD after the full adjustment for shared risk factors and CVD. Numerous studies have shown that CVD stimulates cancer growth. Meijers et al. [58] demonstrated that heart failure (HF) enhanced cancer growth in adenomatous polyposis coli mice. On the other hand, the investigators reported myocardial infarction (MI) accelerates breast cancer growth in mice by increasing the circulating Ly6C^hi^ monocyte levels and recruitment to tumors in MI mice when compared to that in sham mice. However, the depletion of these cells abrogated MI-induced tumor growth [59].

APOE is a major component of chylomicron, LDL-C, HDL-C, and very low density lipoprotein cholesterol (VLDL-C) that facilitates assimilation and lipid transfer [60]. *APOE* shows polymorphism attributable to two SNPs (rs429358 and rs7412), which result in 6 different genotypes [61]. However, the effect of *APOE* polymorphisms on cancer risk is not yet established. Watson et al. reported a case-control study involving 206 colorectal cancer patients and 353 healthy controls from the UK population and found that *APOE* ε2/ε3 was a risk factor of colorectal cancer in men, but not in women. Moreover, ε4 carriers showed no significant difference in this regard [45]. However, a meta-analysis identified ε4 allele as a risk factor for breast cancer susceptibility among Asians [62]. In an American population, the deficiency of ε3 significantly increased the risk of colorectal cancer, especially for people aged > 64 years [37]. A study performed in China Taiwan consisting of 291 breast cancer patients and 148 controls suggested that the incidence of left-side cancer site was greater among ε2 carriers than among ε3 carriers in premenopausal women [63]. DE et al. reported that, when compared with ε3 homozygous, patients with at least one *APOE* ε2 allele showed a significantly increased (60%) risk of gastric cancer [34]. A meta-analysis revealed that *APOE* ε4 allele increases the risk of cancer in Asians [64]. Indeed, the cancer types evaluated in this meta-analysis paper only included breast and colorectal cancers. In the present study, we enrolled all types of solid tumors and found that the *APOE* ε4 allele was an independent risk factor for tumor patients with or without CVD based on the results of logistics analysis, as also confirmed by the above findings.

To the best of our knowledge, this is the first study to identify *APOE* polymorphism in tumors with or without CVD in a southern China population, with the E3/E3 identified as the most common genotype and E4/E4 and E2/E2 as the least common ones in our study population. In tumor patients with or without CVD, the mean age was significantly higher than those of the control subjects (63.33 years versus 61.53 years, *P* < 0.05; 69.89 years versus 61.53 years, *P* < 0.001, respectively). Furthermore, CVD patients also were older (64.96 years versus 61.53 years, *P* < 0.001), indicating that older people are at significantly more risk of tumor or CVD. According to the American Heart Association (AHA) guidelines, individuals of age > 40 years require health-care management with age elevated 10-year risk of ≥ 7.5% [65]. The frequency of the ε4 allele and the E3/E4 genotype was significantly higher among our tumor patients. When compared to the controls, individuals carrying the ε4 allele had an increased tumor risk by 2.14-folds and a risk of tumor + CVD by 2.48 folds (*P* < 0.01). Moreover, gender and SBP acted as independent risk factors (all *P* < 0.01). We also noted that HDL-C acted as an independent protective factor for the development of tumors with or without CVD (*P* < 0.001). The blood lipid profiles largely showed an impact on the relationship between *APOE* polymorphisms and tumors. Kang et al. also reported that the presence of *APOE* ε2 with lower TC significantly increased the risk of gastric cancer [42]. A statistically significant association has been reported between *APOE* ε4 and elevated levels of VLDL-C and TG in colorectal cancer patients [38]. Anand et al. reported that lower HDL-C may be a risk factor for the development of cancer-based on the analyses of the association between *APOE* polymorphism and cancer susceptibility among Asians [48]. Higher serum concentrations of TG and ApoB and lower HDL-C levels were recorded in tumor ε4 allele carriers among the southern China population in the present study; these findings are consistent with those of our past research studies. However, the TC and LDL-C levels increased both in the tumor and CVD groups, which does not conform to past study reports [16]. The reasons for this discrepancy can be explained by the clinical characteristics, different ethnicity, and other related confounding factors. On the other hand, we also found that tumor ε3 allele carriers had a similar change in their lipid profiles.

Nevertheless, this is the first study on the relationship between *APOE* genetic polymorphisms and cancer and CVD in a southern Chinese population. We recognized several limitations in this study. First, the results may have some deviations considering that all types of malignant tumors were assessed in this case-control study. Second, the study was conducted only among Meizhou Chinese people; hence, the findings of other populations warrant further investigation. Third, the sample size selection of this study was not sufficiently large, thereby warranting further genetic studies on *APOE* genetic polymorphisms to elucidate the present findings. Finally, this retrospective study only investigated the relationship between blood lipids and *APOE* polymorphisms and cancer and CVD, but did not include other biochemical or molecular markers, such as inflammatory markers or ncRNAs.

## Conclusions

Our study results suggest that ε4, as well as TG and SBP, are independent risk factors, while HDL-C is a protective factor for the development of tumors with or without CVD in southern China. *APOE* polymorphisms may hence serve as a guide for identifying individuals at risk of tumor so as to design precise preventive strategies and therapies.

## Data Availability

The datasets generated during the current study are not publicly available yet, due to privacy concerns and ongoing additional research. Data can be made available for peer review on reasonable request through contacting the corresponding author.

## Abbreviations

CVD: Cardiovascular disease
APOE: Apolipoprotein E
AD: Age-related Alzheimer’s disease
T2DM: Type 2 diabetes mellitus
SBP: Systolic blood pressure
DBP: Diastolic blood pressure
TC: Total cholesterol
TG: Triglyceride
LDL-C: Low-density lipoprotein cholesterol
HDL-C: High-density lipoprotein cholesterol
OR: Odds ratio
CI: Confidence interval
HWE: Hardy-Weinberg equilibrium
CA 15-3: Cancer antigen 15-3
CA 19-9: Cancer antigen 19-9
CEA: Carcinoembryonic antigen
CYFPRA 21-1: Cytokeratin fragment 21-1
AFP: Alpha-fetoprotein
